# Whole-Genome Sequences of *Staphylococcus aureus* ST398 Strains of Animal Origin

**DOI:** 10.1128/genomeA.00689-13

**Published:** 2013-09-26

**Authors:** David Hernandez, Nathalie van der Mee-Marquet, Jan Kluytmans, Pierre-Yves Donnio, Roland Quentin, Anna-Rita Corvaglia, Patrice François

**Affiliations:** Genomic Research Laboratory, University of Geneva Hospitals, Geneva, Switzerlanda; Service de Bactériologie et Hygiène, Centre Hospitalier Régional Universitaire, Tours, Franceb; Réseau des Hygiénistes du Centre, Centre Hospitalier Universitaire, Tours, Francec; Laboratory for Microbiology and Infection Control, Amphia Hospital, Breda, and VU University Medical Center, Amsterdam, The Netherlandsd; Service de Bactériologie et Hygiène Hospitalière, Centre Hospitalier Universitaire, Rennes, Francee; EA1254 Microbiologie-Risques Infectieux, Université de Rennes 1, Rennes, Francef

## Abstract

*Staphylococcus aureus* sequence type 398 (ST398) was originally associated with animal infections. We announce the complete genome sequences of two ST398 methicillin-susceptible *S. aureus* strains from the livestock environment. These genome sequences assist in the characterization of interesting ST398 features relying on host tropism and epidemiological settings*.*

## GENOME ANNOUNCEMENT

*Staphylococcus aureus* sequence type 398 (ST398) was originally described in livestock (1) but is currently reported as the causative agent of severe infections in humans (2). We recently reported the emergence of a specific subpopulation of ST398 showing specific bacteriophage content and appearing to be adapted to humans (2). We report here the genome sequences of two *S. aureus* strains colonizing humans in an animal environment.

*S. aureus* strains S1 and S130 were recovered from cows and pigs, respectively. S1 and S130 were isolated from feces samples during the course of epidemiological studies from healthy farmers conducted in 2008 in France and 2010 in the Netherlands, respectively. Purified genomic DNA was subjected to whole-genome shotgun sequencing by using a MiSeq system (Illumina, Inc). Following fragmentation, end reparation, and sample tagging, the sequencer produced 10.0 million 100-bp paired reads for each strain, yielding appreciable and identical coverages of around 360×. Assembly was performed using Edena 3.0 (3) and resulted in 60 and 42 contigs for strains S1 and S130, respectively. The larger contigs of 475,000 bp for strain S1 and 567,000 bp for strain S130 were assembled. The overall assembly values were satisfactory (strain S1 sum, 2.82 Mbp, and N_50_, 241,000 bp; strain S130 sum, 2.78 Mbp, and N_50_, 140,000 bp). In strains S1 and S130, a total of 2,618 and 2,595 predicted coding sequences (CDSs), respectively, were detected by Rapid Annotations using Subsystems Technology (RAST) (4). Using Cd-hit (5), we found that the majority of genes (*n* = 2,470) were common to both strains (identity, >80%). More than 53% of the genes were assigned to specific subsystem categories by RAST (4). In addition to CDSs, RAST identified 78 structural genes, including 59 tRNA and 19 rRNA genes, for the chromosome of S1 and 76 structural genes, including 59 tRNA and 17 rRNA genes, for the chromosome of S130. Note that the assembly results showed that strain S130 harbors one 9.0-kb circular plasmid in 3 copies/cell, whereas S1 harbors 4 circular plasmids. The sizes of these plasmids are 4.90, 3.99, 2.75, and 2.46 kb with copy numbers/cell of around 10, 12, 20, and 8, respectively.

The two *S. aureus* genomes are highly similar in terms of annotations and contain known virulence factors, such as genes for enterotoxin and hemolysins, as well as 30 open reading frames (ORFs) from a prophage origin. Annotation allowed for the detection of numerous bacterial adhesins that allow interaction with host tissues and also numerous resistance determinants. Interestingly, both strains are missing the type IV restriction system but harbor a type I restriction-modification system with a sequence similar to that of functional systems in the reference isolates.

We conclude that *S. aureus* shows important genome features specific to its host and epidemiological settings.

### Nucleotide sequence accession numbers.

The whole-genome sequences of *S. aureus* S1 and S130 were deposited in DDBJ/EMBL/GenBank under accession no. AUPS00000000 and AUPT00000000, respectively.

## References

[1] Witte W Strommenger B Stanek C Cuny C 2007 Methicillin-resistant *Staphylococcus aureus* ST398 in humans and animals, Central Europe. Emerg. Infect. Dis. 13:255–258 17479888 10.3201/eid1302.060924 PMC2725865

[2] Mee-Marquet N Corvaglia AR Valentin AS Hernandez D Bertrand X Girard M Kluytmans J Donnio PY Quentin R Francois P 2013 Analysis of prophages harbored by the human-adapted subpopulation of *Staphylococcus aureus* CC398. Infect. Genet. Evol. 18:299–308 23770143 10.1016/j.meegid.2013.06.009

[3] Hernandez D François P Farinelli L Osterås M Schrenzel J 2008 *De novo* bacterial genome sequencing: millions of very short reads assembled on a desktop computer. Genome Res. 18:802–809 18332092 10.1101/gr.072033.107 PMC2336802

[4] Aziz RK Bartels D Best AA DeJongh M Disz T Edwards RA Formsma K Gerdes S Glass EM Kubal M Meyer F Olsen GJ Olson R Osterman AL Overbeek RA McNeil LK Paarmann D Paczian T Parrello B Pusch GD Reich C Stevens R Vassieva O Vonstein V Wilke A Zagnitko O 2008 The RAST server: rapid annotations using subsystems technology. BMC Genomics 9:75. 10.1186/1471-2164-9-75 18261238 PMC2265698

[5] Li W Godzik A 2006 Cd-hit: a fast program for clustering and comparing large sets of protein or nucleotide sequences. Bioinformatics 22:1658–1659 16731699 10.1093/bioinformatics/btl158

